# Caesarean section and severe upper and lower respiratory tract infections during infancy: Evidence from two UK cohorts

**DOI:** 10.1371/journal.pone.0246832

**Published:** 2021-02-16

**Authors:** Neora Alterman, Jennifer J. Kurinczuk, Maria A. Quigley

**Affiliations:** 1 National Perinatal Epidemiology Unit, Nuffield Department of Population Health, University of Oxford, Oxford, United Kingdom; 2 NIHR Policy Research Unit in Maternal Health and Care, National Perinatal Epidemiology Unit, Nuffield Department of Population Health, University of Oxford, Oxford, United Kingdom; Centre Hospitalier Universitaire Vaudois, FRANCE

## Abstract

**Background:**

Several studies have reported that birth by caesarean section is associated with increased risk of lower respiratory tract infections in the child, but it is unclear whether this applies to any caesarean section or specifically to planned caesareans. Furthermore, although infections of the upper respiratory tract are very common during childhood, there is a scarcity of studies examining whether caesarean is also a risk factor for this site of infection.

**Methods:**

We obtained data from two UK cohorts: the Millennium Cohort Study (MCS) and linked administrative datasets of the population of Wales through the Secure Anonymised Information Linkage (SAIL) databank. The study focused on term-born singleton infants and included 15,580 infants born 2000–2002 (MCS) and 392,145 infants born 2002–2016 (SAIL). We used information about mode of birth (vaginal delivery, assisted vaginal delivery, planned caesarean and emergency caesarean) from maternal report in the MCS and from hospital birth records in SAIL. Unplanned hospital admission for lower respiratory tract infection (LRTI) was ascertained from maternal report in the MCS and from hospital record ICD codes in SAIL. Information about admissions for upper respiratory tract infection (URTI) was available from SAIL only. Cox regression was used to estimate hazard ratios for each outcome and cohort separately while accounting for a wide range of confounders. Gestational age at birth was further examined as a potential added, indirect risk of planned caesarean birth due to the early delivery.

**Findings:**

The rate of hospital admission for LRTI was 4.6 per 100 child years in the MCS and 5.9 per 100 child years in SAIL. Emergency caesarean was not associated with LRTI admission during infancy in either cohort. In the MCS, planned caesarean was associated with a hazard ratio of 1.39 (95% CI 1.03, 1.87) which further increased to 1.65 (95% CI 1.24, 2.19) when gestational age was not adjusted for. In SAIL, the adjusted hazard ratio was 1.10 (95% CI 1.05, 1.15), which increased to 1.17 (95% CI 1.12, 1.22) when gestational age was not adjusted for. The rate of hospital admission for URTI was 5.9 per 100 child years in SAIL. Following adjustments, emergency caesarean was found to have a hazard ratio of 1.09 (95% CI 1.05, 1.14) for hospital admission for URTI. Planned caesarean was associated with a hazard ratio of 1.11 (95% CI 1.06, 1.16) which increased to 1.17 (95% CI 1.12, 1.22) when gestational age was not adjusted for.

**Conclusions:**

The risk of severe LRTIs during infancy is moderately elevated in infants born by planned caesarean compared to those born vaginally. Infants born by any type of caesarean may also be at a small increased risk of severe URTIs. The estimated effect sizes are stronger if including the indirect effect arising from planning the caesarean birth for an earlier gestation than would have occurred spontaneously. Further studies are needed to confirm these results.

## Introduction

Caesarean section rates have risen steeply in recent years, particularly in high and middle-income countries [1]. In England, for example, the caesarean rate has increased more than 3 fold in less than four decades–from 9% in 1980 [2] to 28.4% in 2018 [3]. An increase has taken place both for planned (‘elective’) caesarean sections and for unplanned, ‘emergency’ surgery. Because of this increase, the World Health Organization issued a statement in 2015 about rates of caesarean section [4] in which they called for further research to better understand the health effects of caesareans, including on longer-term child outcomes.

Acute respiratory infections are common during childhood. These infections include those manifesting in the lower respiratory tract such as bronchiolitis or pneumonia, and those of the upper respiratory tract such as the common cold, tonsillitis and laryngitis. The majority of respiratory infections are treated in primary care, however, some require hospitalisation. In Wales, for example, approximately 11% of infants were reported to require hospital admission for a respiratory infection by age 1 year [5]. These admissions were due to an infection of the upper respiratory tract in 45% of cases and due to bronchiolitis in 44% [5].

Caesarean section is a risk factor for hospital admission for lower respiratory infections (LRTIs), both during infancy [6–9] and later in childhood [10–13]. When examining elective and emergency caesareans separately, the available evidence suggests that elective caesareans are associated with an increased risk of infection [7–11], while the evidence for emergency caesarean is less conclusive [7, 8, 11]. Some studies have examined hospital admissions for any respiratory morbidity combined and found caesarean section to increase the risk of unplanned hospitalisation [5, 14]. However, only a single study that we are aware of has specifically examined the effect of mode of birth on severe upper respiratory tract infections (URTIs) [13]. This large international study identified a small increase in the risk for hospital admission for URTI in infants and preschool children born by elective or emergency caesarean section, with risks slightly higher following elective caesarean. Study findings relating to hospital admission due to LRTI or other types of infection were of a similar pattern [13].

In addition to mode of birth, other perinatal factors are also associated with respiratory morbidity. Gestational age at birth is an independent risk factor for childhood infections and respiratory illness [5, 15–17]. The severity of neonatal respiratory morbidity is inversely related to gestational week at time of caesarean [18]. Yet, despite this evidence, studies focusing on mode of birth and childhood respiratory infections have not examined the effect arising indirectly from the early delivery when a caesarean is planned [19]. Furthermore, although not being breastfed is also associated with infant infection [20, 21], most prior studies have not accounted for this factor.

The aim of the current study was to further elucidate whether birth by planned or emergency caesarean section is associated with increased risk of hospital admission due to LRTI or URTI in infancy.

## Methods

The study used anonymised data from two birth cohorts analysed separately: the longitudinal UK Millennium Cohort Study and a birth cohort defined through linkage of routinely collected data for the population of Wales.

### Data sources

#### Millennium Cohort Study

The Millennium Cohort Study (MCS) is a nationally representative longitudinal study of babies born between September 2000 and January 2002 that were alive and living in the UK at age 9 months. The sample does not include babies who died prior to this age, but these constituted ~0.5% of all births [22]. The MCS is a stratified cluster sample by electoral ward, with oversampling of ethnic minorities and disadvantaged areas, ensuring adequate representation of responses. A total of 18,818 infants were recruited from 72% of eligible families [23]. The first survey took place when most infants were 9–10 months of age. The infant’s mother was usually the main respondent and information about the family, the birth and the infant’s health was collected [24].

#### SAIL–Secure Anonymized Information Linkage Databank

The Secure Anonymized Information Linkage Databank (SAIL) is a data repository developed at Swansea University that stores anonymized data for the population of Wales. This databank hosts several health and administrative datasets of person-level data. Individuals can be linked between the various datasets using a unique Anonymous Linking Field (ALF) encrypted code, which is generated based on NHS number or personal identifiers [25, 26].

We defined a cohort of infants born between January 2002 and July 2016 to mothers resident in Wales as ascertained from the National Child Community Health Database (NCCHD) [27] (N = 490,826). The NCCHD is the all-Wales child health surveillance dataset and includes records of all children born, resident or treated in Wales. The records include a maternal ALF code as well as information about perinatal factors. The hospital birth record of these infants was derived from the Patient Episode Dataset for Wales (PEDW), which includes all inpatient and day case admissions in NHS Wales and those of Welsh residents in English hospitals. The relevant birth hospital admission was identified by matching on maternal ALF code with the further requirement that date of admission spanned the infant’s week of birth and that the admission record includes an Operating Procedure Codes (OPCS-4) indicating a birth. Information about maternal health was derived from diagnosis codes (ICD-10) from the birth admission. The infants identified were further linked to the Welsh Demographic Dataset (WDS) [28], a dataset including all residents registered with a General Practice (GP) in Wales, from which quintiles of the Welsh Index of Multiple Deprivation (WIMD) based on area of residency were derived, as well as information on time of emigration from Wales. Infants were also matched to the Annual District Death Extract (ADDE) [29] to derive date of death and to the Congenital Anomaly Register and Information Service for Wales (CARIS) [30] from which those with severe anomalies or genetic abnormalities were identified. The infants eligible to be included in our defined cohort were those live born and with a matching PEDW delivery record and WDS record that indicates being born to a Welsh resident (n = 442,565).

### Inclusion criteria

**Fig 1:**
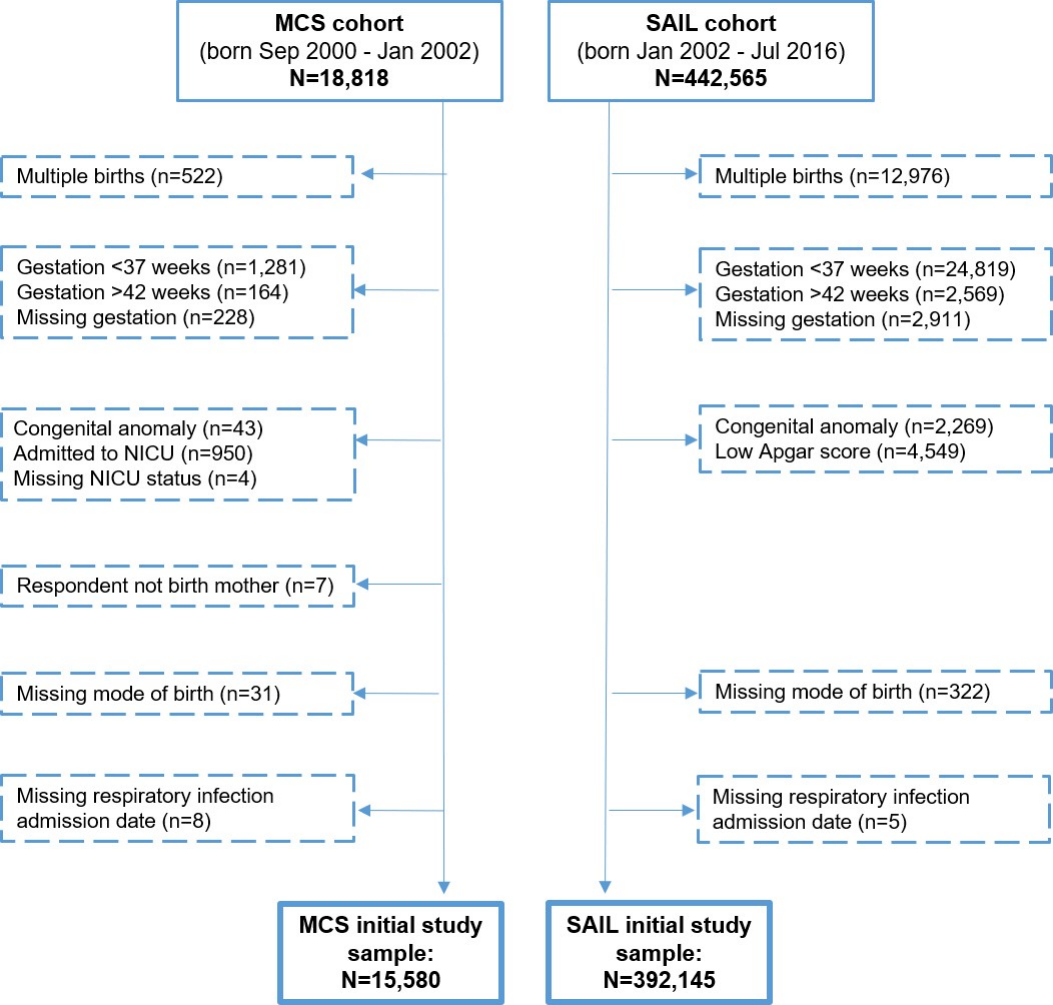
Infants included and excluded from MCS and SAIL cohorts.

Our focus was on healthy infants from birth, therefore the study included singleton infants born alive at term (37–42 gestational weeks) excluding those with a major health problem immediately following the birth (Fig 1). A major health problem was defined as a serious congenital or genetic abnormality (S1 Box in S1 File) and in the MCS also as report of neonatal special medical care and in SAIL also as low Apgar score (0–2 at 1 minute or 0–6 at 5 minutes).

### Exposure–mode of birth

Mode of birth was categorised as vaginal delivery, assisted vaginal delivery, planned caesarean section or emergency caesarean section (Table 1). In the MCS, mode of birth was ascertained from the mother’s report, which is known to be in good agreement with hospital records [31]. In the SAIL cohort, mode of birth was gathered from the birth admission operating procedure codes. When more than a single mode of birth was recorded (n = 115 in MCS and n = 293 in SAIL), the more highly medically interventional method was assigned [32].

**Table 1:** Definitions of exposure—mode of birth.

**Mode of birth classification**	**MCS**	**SAIL**
	Maternal report	Operating procedure codes
**Vaginal delivery (VD)**	Normal delivery	R24 Normal delivery
	Waterbirth	R23 Cephalic vaginal delivery with abnormal presentation of head at delivery without instrument
**Assisted vaginal delivery (Assisted VD)**	Forceps	R19 Breech extraction delivery
	Vacuum	R20 Other breech delivery
	Assisted breech	R21 Forceps cephalic delivery
	Other assisted delivery	R22 Vacuum delivery
**Planned caesarean section (Planned CS)**	Planned caesarean	R17 Elective caesarean delivery
**Emergency caesarean section (Emergency CS)**	Emergency caesarean	R18 Other caesarean delivery
**Missing**	Refuse to answer, not known, irrelevant or non-codable	R25 Other method of delivery

### Outcomes–hospital admission for lower or upper respiratory tract infection

The study outcomes were hospital admission in infancy for lower respiratory infection or for upper respiratory infection. In the MCS, this was collected through mother’s report of hospital admissions due to ‘chest infection or pneumonia’ selected from a pre-defined list, together with the child’s age in months at admission. Almost all MCS sample infants (97%) were followed-up for at least 9 months.

In the SAIL cohort, information about outcomes was collected from hospital admissions recorded in PEDW. The outcomes were defined as an emergency (unplanned) admission during infancy with a diagnosis code indicating a lower respiratory tract infection (A37, J10-22) or an upper respiratory tract infection (J00-J06) (Table 2). Admissions were available from January 2002 to February 2017. There was complete follow-up until the 1^st^ birthday for 98% of the SAIL cohort.

**Table 2:** Definitions of outcomes–lower and upper respiratory tract infections.

MCS	SAIL
Maternal report	Diagnosis codes
**Lower respiratory tract infection (LRTI)**	
Chest infection or pneumonia	A37—Whooping cough
	J10—Influenza due to identified influenza virus
	J11—Influenza, virus not identified
	J12—Viral pneumonia, not elsewhere classified
	J13—Pneumonia due to Streptococcus pneumoniae
	J14—Pneumonia due to Haemophilus influenzae
	J15—Bacterial pneumonia, not elsewhere classified
	J16—Pneumonia due to other infectious organisms, not elsewhere classified
	J17—Pneumonia in diseases classified elsewhere
	J18—Pneumonia, organism unspecified
	J20—Acute bronchitis
	J21—Acute bronchiolitis
	J22—Unspecified acute lower respiratory infection
**Upper respiratory tract infection (URTI)**	
Not available	J00—Acute nasopharyngitis [common cold]
	J01—Acute sinusitis
	J02—Acute pharyngitis
	J03—Acute tonsillitis
	J04—Acute laryngitis and tracheitis
	J05—Acute obstructive laryngitis [croup] and epiglottitis
	J06—Acute upper respiratory infections of multiple/unspecified sites

### Explanatory variables

Potential explanatory variables were ascertained through parental report in the MCS and through information derived from the NCCHD, WDS or PEDW delivery record in SAIL (Table 3).

**Table 3:** Explanatory variables available for the analyses.

Explanatory variable	MCS	SAIL
		[Dataset]
**Socio-demographic variables**		
Mother’s age in years	<20, 20–29, 30–39, ≥40	<18, 18–19, 20–24, 25–29, 30–34, 35–39, ≥40
		[NCCHD]
Mother’s marital status	married, cohabiting, single	–
Mother’s education level	university degree, ‘A levels’, lower than ‘A levels’, other and overseas, no formal qualifications	–
Socioeconomic status (last known occupation of the mother or her partner, if higher)	managerial or professional, intermediate, routine or manual, never worked or long-term unemployed	–
Area deprivation quintile (Welsh Index of Multiple Deprivation—WIMD)	–	1^st^ (most deprived) - 5^th^ (least deprived)
		[WDS]
Ethnicity	white, other	white, mixed, black Indian/Pakistani/Bangladeshi, other, missing
		[NCCHD]
***Maternal and perinatal variables***		
Sex	male, female	male, female
		[NCCHD]
Firstborn	yes, no	yes, no
		[NCCHD+PEDW]*
Maternal asthma / atopic disease	eczema, hay fever or asthma	yes (ICD code J45 or J46 in any of mother’s delivery records for children included in sample), no
		[PEDW]
Maternal diabetes	yes, no	yes (ICD codes O24, E10-E14), no
		[PEDW]
Maternal hypertensive condition	yes, no	yes (ICD codes O10-O16, I10-I15), no
		[PEDW]
Birthweight**	weight in Kg	weight in Kg
		[NCCHD]
Gestational age birth**	37–38, 39–40, 41, 42	37, 38, 39, 40, 41, 42
		[NCCHD]
Maternal smoking	non-smoker, ex-smoker, relapsed smoker, postnatal quitter, took up smoking postnatally, smoker	non-smoker, gave up in pregnancy, smokes 1–9 a day, smokes ≥10 a day, missing
		[NCCHD]
Breastfeeding	any breastfeeding per month of follow-up (i.e. time-dependent) [52]	any breastfeeding (at birth or at 8 weeks), none, missing
		[NCCHD]
Year of birth	–	2002–2016
		[NCCHD]
Season of birth	–	January-March, April-June,
		July-September, October-December
		[NCCHD]

* coded from NCCHD data on week of birth of child and sibling records, previous live and stillbirth of cohort child and sibling records and from ICD codes in PEDW delivery record indicating a prior birth

** In the MCS, gestational age was calculated by comparing mother’s report of her due date with the actual date of birth, a variable which has been previously validated with linked hospital birth records. In both cohorts, observations with implausible birthweight for gestational age were recoded to missing [53]

### Statistical analysis

Hazard ratios for each mode of birth compared with vaginal delivery (the reference group) were estimated using Cox regression analysis. In the MCS, follow-up began at time of discharge from hospital of birth (median 2 days) and in SAIL follow-up began at time of birth. Follow-up ended at the first hospital admission for LRTI or URTI or was censored in the MCS at age at interview (range 6–12 months; average 9.2 months) and in SAIL at time of emigration from Wales, death, or February 2017. Statistical analyses of the MCS accounted for the complex survey design as well as weighting for non-response using Stata version 13 ‘survey commands’. Statistical analyses of the SAIL cohort accounted for within-family clustering since most infants had a sibling in the cohort (60%). The Cox regression assumption of constant hazard ratios was verified in the MCS using log-log plots and interaction with time and in SAIL using log-log plots and assessing whether hazard ratios are substantially different when follow-up is split into two time periods.

We selected variables to be included in multivariable models as follows. In the MCS, sex was included *a priori* and the association of other variables found to be associated (P<0.1) with the exposure and the outcome was examined in a two stage process. Initially, socio-demographic variables and maternal-perinatal variables (Table 3) were included separately in a multivariable model and then statistically significant (P<0.1) variables from each model were combined and only those that significantly improved the fit of the data were included in the final model. In the SAIL cohort all models were adjusted for year of birth and multivariable models were adjusted for those covariates listed in Table 3 that were associated (P<0.1) with both exposure and outcome.

#### Missing data

In the MCS, missing data about explanatory variables was minimal and therefore complete case analysis was used. In the SAIL cohort most variables had <10% missing values but the proportion was higher for smoking (60%), breastfeeding (15%) and ethnicity (11%) and therefore a category of ‘missing’ was included for these variables. Complete case analysis and multiple imputation were used in sensitivity analyses (S1 Text in S1 File).

#### Effect of gestational age

In the case of planned caesarean section (but less commonly in emergency caesarean), gestational age at birth is the result of the planned mode of birth. We aimed to quantify the risk of respiratory infection due to caesarean section while taking account of the potential added risk from an earlier delivery at early term (37–38 weeks). Final models were therefore repeated without adjustment for gestational age.

### Sensitivity analyses

Wheezing is a common symptom of respiratory infection, therefore a sensitivity analysis was carried out in the MCS including ‘wheezing or asthma’ in the outcome definition. To address the possibility of reverse causality of a prenatal infection leading both to need for a caesarean birth and to later infant infection, we carried out an additional sensitivity analysis in the MCS excluding infants with a report of infection in the perinatal period. Further sensitivity analyses limiting to observations where mode of birth had been validated using an additional data source (S2 Text and S2 Box in S1 File) were also carried out in both cohorts.

### Ethics

The MCS was granted ethics approval from Multi-Centre Research Ethics Committees [33]. Permission to use SAIL data was granted from the databank’s Information Governance Review Panel.

## Results

### Sample characteristics

The study included 15,580 infants for the MCS analyses and 392,145 infants for the SAIL analyses. Table 1 presents the distribution of mode of birth in the two cohorts. In the MCS, there were 71.1% vaginal deliveries, 10.1% assisted vaginal deliveries, 8.7% planned caesareans and 10.1% emergency caesareans. In the SAIL cohort there were 63.9% vaginal deliveries, 11.8% assisted vaginal deliveries, 11.0% planned caesareans and 13.3% emergency caesareans. When comparing the SAIL births taking place in 2002 (the most proximate year to when MCS children were born) to Welsh births of the MCS, the distribution of mode of birth was similar (Table 4).

**Table 4:** Distribution of mode of birth in MCS and SAIL cohorts.

Proportion	VD (%)	Assisted VD (%)	Planned CS (%)	Emergency CS (%)	Total (%)
Total MCS sample*	71.1	10.1	8.7	10.1	100
**Total SAIL sample**	63.9	11.8	11.0	13.3	100
MCS Welsh births only**	68.7	10.1	10.0	11.3	100
**SAIL 2002 births only**	67.6	9.6	10.7	12.1	100

* Percentages weighted using total UK weights

** Percentages weighted using country-specific weights

The characteristics of both cohorts were strongly associated with mode of birth (Table 5; P<0.001 for all variables except P<0.025 for maternal atopy). Mothers who had given birth by caesarean section (both planned and emergency) tended to be older, married, more highly educated and of higher socioeconomic status or living in a more affluent area compared to mothers who had a vaginal delivery. These mothers had more complications of pregnancy such as diabetes or hypertensive conditions but smoked less than those who had given birth vaginally. The infants born by planned caesarean were less likely to be the firstborn child compared to those born vaginally, while those born by emergency caesarean were more likely to be firstborn. The group of infants born by planned caesarean were also more likely to be born at early term (37–38 weeks), while the emergency caesarean group were more likely to be born at late term (41 weeks) and post term (42 weeks). See also S1 Table in S1 File.

**Table 5:** Characteristics of SAIL and MCS cohorts by mode of birth.

	MCS*					**SAIL**				
	VD	Assisted	Planned	Emergency	P-value	VD	Assisted VD	Planned	Emergency CS	P-value
	%**	VD %	CS %	CS %		%	%	CS %	%	
	n = 11,077	n = 1,528	n = 1,381	n = 1,594		n = 250,740	n = 46,086	n = 43,231	n = 52,088	
***Socio-demographics***										
**Maternal age, mean (SD)**	28.4 (5.8)	28.7 (5.5)	31.1 (5.2)	29.8 (5.7)	<0.001	27.5 (5.9)	27.8 (5.9)	30.7 (5.7)	28.7 (6.0)	<0.001
**Marital status**					<0.001					
Married	58.6	62.6	71.8	64.4		–	–	–	–	
Cohabiting	25.9	26.4	18.7	24.0		–	–	–	–	
Single	15.5	11.1	9.5	11.6		–	–	–	–	
**Education level**					<0.001					
Higher	30.4	41.0	37.5	40.3		–	–	–	–	
Intermediate	14.6	15.1	11.9	14.0		–	–	–	–	
Lower	38.6	35.4	37.4	33.8		–	–	–	–	
Other academic / vocational	2.6	2.2	2.1	2.7		–	–	–	–	
None of these	13.8	6.5	11.0	9.2		–	–	–	–	
**Socioeconomic class**					<0.001					
Managerial/Professional	42.5	53.9	48.2	53.7		–	–	–	–	
Intermediate	19.8	18.6	22.8	18.4		–	–	–	–	
Routine/Manual	32.5	24.4	26.3	24.4		–	–	–	–	
Unemployed	5.2	3.1	2.7	3.6		–	–	–	–	
**Area deprivation quintile**										<0.001
1^st^ (most deprived)	–	–	–	–		27.7	21.9	22.9	23.2	
2^nd^	–	–	–	–		22.2	21.1	21.2	21.6	
3^rd^	–	–	–	–		19.1	19.7	19.4	20.1	
4^th^	–	–	–	–		16.3	18.3	17.2	18.3	
5^th^ (least deprived)	–	–	–	–		14.8	19.1	19.2	16.8	
**White ethnicity**	86.7	91.2	89.2	85.9	<0.001	91.7	91.9	92.5	91.3	<0.001
***Maternal-perinatal***										
**Male**	49.5	55.1	47.9	55.3	<0.001	49.7	54.0	50.6	55.3	<0.001
**Firstborn**	34.1	78.2	23.1	68.6	<0.001	35.4	75.8	20.5	65.5	<0.001
**Maternal asthma**	–	–	–	–		5.3	5.5	6.5	6.1	<0.001
**Maternal atopy**	42.2	46.2	40.7	40.7	0.025	–	–	–	–	
**Diabetes**	1.5	1.7	4.6	2.3	<0.001	1.6	2.0	4.8	3.6	<0.001
**Hypertension**	6.0	8.5	7.2	13.0	<0.001	4.4	8.3	4.1	10.4	<0.001
**Birthweight in kg**, mean (SD)	3.4 (0.5)	3.5 (0.5)	3.4 (0.5)	3.5 (0.5)	<0.001	3.4 (0.5)	3.5 (0.5)	3.5 (0.5)	3.5 (0.5)	<0.001
**Gestational age**					<0.001					<0.001
37–38 (early term)	17.7	12.1	54.2	17.8		16.7	13.4	34.0	18.5	
39–40 (full term)	56.3	53.3	39.3	46.8		56.5	51.4	58.8	44.5	
41 (late term)	22.6	29.5	4.9	28.9		23.1	28.3	5.7	28.2	
42 (post term)	3.5	5.2	1.7	6.6		3.7	7.0	1.4	8.8	
**Maternal smoking**					<0.001					<0.001
Non-smoker	65.2	67.5	69.9	70.7		74.5	81.5	82.2	80.9	
Gave-up in pregnancy	12.0	15.7	11.7	14.5		3.7	4.1	2.6	4.0	
Smoker	22.8	16.8	18.4	14.9		21.8	14.4	15.2	15.1	
**Any breastfeeding**	68.5	75.2	69.5	74.6	<0.001	56.9	63.9	56.8	61.8	<0.001

* Percentages in MCS are weighted.

** All MCS and SAIL percentages are calculated from non-missing observations. Missing values are <10% of total observations for all variables apart from the following in SAIL: 59.8% for smoking, 15.3% for breastfeeding, 11.2% for ethnicity.

### Hazard ratios of hospital admission for LRTI

Overall, 3.4% (95% CI 3.1, 3.8) of infants were admitted at least once for LRTI in the MCS sample (574 cases) and 5.6% (95% CI 5.6, 5.7) in the SAIL sample (22,154 cases). To aid comparability, the proportion of infants born in 2002 and admitted up to age 9.2 months in SAIL was examined. This proportion was found to be 4.0% (95% CI 3.7, 4.2), similar to 3.4% in the MCS. The rate of admission peaked at the 2^nd^-3^rd^ month of life and later declined (S1 Fig in S1 File). In the SAIL sample, admissions were predominantly for acute bronchiolitis (J21; 83% of events) and much less frequently for unspecified LRTI (J22; 9% of events), pneumonia with unspecified organism (J18; 4% of events) or other indications. In 95% of cases, LRTI was the primary indication for admission.

The rate of hospital admission for LRTI was 4.6 per 100 child-years in the MCS and 5.9 per 100 child-years in the SAIL cohort (Table 6). In the crude model of both cohorts, infants born by assisted vaginal delivery and emergency caesarean section had a lower hazard of admission compared to those born by vaginal delivery. Following adjustment for confounders and other explanatory variables, these modes of birth were not associated with LRTI hospital admission. Infants born by planned caesarean section were more likely to be admitted to hospital (51% increased risk in the MCS and 15% in SAIL), and this increase in risk remained after accounting for other variables: adjusted HR = 1.39 (95% CI 1.03 to 1.87) in the MCS and adjusted HR = 1.10 (95% CI 1.05 to 1.15) in SAIL. The findings from SAIL using complete case analysis and from multiple imputation were similar to the main results (S2 Table in S1 File).

**Table 6:** Association between mode of birth and hospital admission for LRTIs and URTIs in the MCS and SAIL cohorts.

	**Hospital admission for**										**Hospital admission for**				
	Lower Respiratory Tract Infection										Upper Respiratory Tract Infection				
	MCS					SAIL					SAIL				
	No. of events	Child-years	Rate per 100 child-years	Crude hazard ratio	Adjusted hazard ratio *	No. of events	Child-years	Rate per 100 child-years	Crude hazard ratio **	Adjusted hazard ratio ***	No. of events	Child-years	Rate per 100 child-years	Crude hazard ratio **	Adjusted hazard ratio ****
				(95% CI)	(95% CI)				(95% CI)	(95% CI)				(95% CI)	(95% CI)
				P-value	P-value				P-value	P-value				P-value	P-value
				n = 15,580	n = 15,531				n = 392,145	n = 364,651				n = 392,145	n = 364,654
Overall	574	11,664	4.6			22,154	374,489	5.9			22,278	377,422	5.9		
VD	420	8,308	4.6	1.00	1.00	14,697	239,134	6.2	1.00	1.00	14,073	241,384	5.8	1.00	1.00
Assisted VD	43	1,181	3.8	0.82 (0.57,1.19)	1.18 (0.79, 1.75)	2,024	44,348	4.6	0.73 (0.69, 0.76)	0.97 (0.93, 1.02)	2,495	44,425	5.6	0.95 (0.91, 0.99)	1.03 (0.98, 1.07)
				P = 0.301	P = 0.420				P<0.001	P = 0.304				P = 0.024	P = 0.292
Planned CS	61	999	7.0	1.51 (1.13, 2.03)	1.39 (1.03, 1.87)	2,942	41,005	7.2	1.15 (1.11, 1.20)	1.10 (1.05, 1.15)	2,717	41,481	6.6	1.12 (1.07, 1.16)	1.11 (1.06, 1.16)
				P = 0.006	P = 0.030				P<0.001	P<0.001				P<0.001	P<0.001
Emergency CS	50	1,176	3.7	0.83 (0.58, 1.20)	1.14 (0.79, 1.65)	2,491	50,002	5.0	0.80 (0.77, 0.84)	1.03 (0.98, 1.08)	2,993	50,132	6.0	1.02 (0.98, 1.06)	1.09 (1.05, 1.14)
				P = 0.328	P = 0.494				P<0.001	P = 0.244				P = 0.326	P<0.001

* Adjusted for maternal age, firstborn, infant’s sex, maternal smoking, gestational age and breastfeeding per month of follow-up

** Adjusted for year of birth

***Adjusted for maternal age, area deprivation quintile, firstborn, smoking, maternal asthma, hypertensive conditions, infant’s sex, ethnicity, gestational age, birthweight, breastfeeding, season and year of birth

**** Adjusted for maternal age, area deprivation quintile, firstborn, smoking, maternal asthma, infant’s sex, ethnicity, gestational age, birthweight, breastfeeding, season and year of birth

Findings from further sensitivity analyses were consistent with the main results. These included using validated mode of birth data in both cohorts (S3 Table in S1 File), and in the MCS—excluding infants with a perinatal infection (S4 Table in S1 File) and using a broader definition of the outcome (S5 Table in S1 File).

### Hazard ratios of hospital admission for URTI

Hospital admission for URTI was available solely in the SAIL cohort. Overall, the proportion of infants who were admitted at least once for URTI was 5.7% (95% CI 5.6 to 5.8) (22,278 cases). The rate of admission increased towards the infant’s 1^st^ birthday (S2 Fig in S1 File). Admissions were predominantly for acute URTI of multiple or unspecified sites (J06; 72% of events) and much less frequently due to tonsillitis (J03; 12% of events), croup (J05; 11% of events) or other indications. The URTI was the primary indication for admission in 91% of cases. This group of infants had little overlap with the group admitted for LRTI—only 3% of infants admitted for URTI had a diagnosis code for LRTI too.

The rate of URTI hospital admission was 5.9 per 100 child-years (Table 6). Infants born by assisted vaginal delivery did not have a different risk of admission in comparison to the risk of infants born vaginally, either before or after adjustments. Infants born by emergency caesarean had a similar risk to the vaginal delivery group, however after adjustment for other variables the excess risk slightly increased (adjusted HR = 1.09; 95% CI 1.05 to 1.14). Infants born by planned caesarean had a small increased risk (12%) of hospital admission for URTI, and this elevated risk remained the same following adjustment (adjusted HR = 1.11 95% CI 1.06 to 1.16). Results from multiple imputation and complete case analysis were in agreement with the main findings (S2 Table in S1 File) as well as those restricted to validated observations (S3 Table in S1 File).

### Indirect effect of mode of birth through earlier delivery

**Fig 2:**
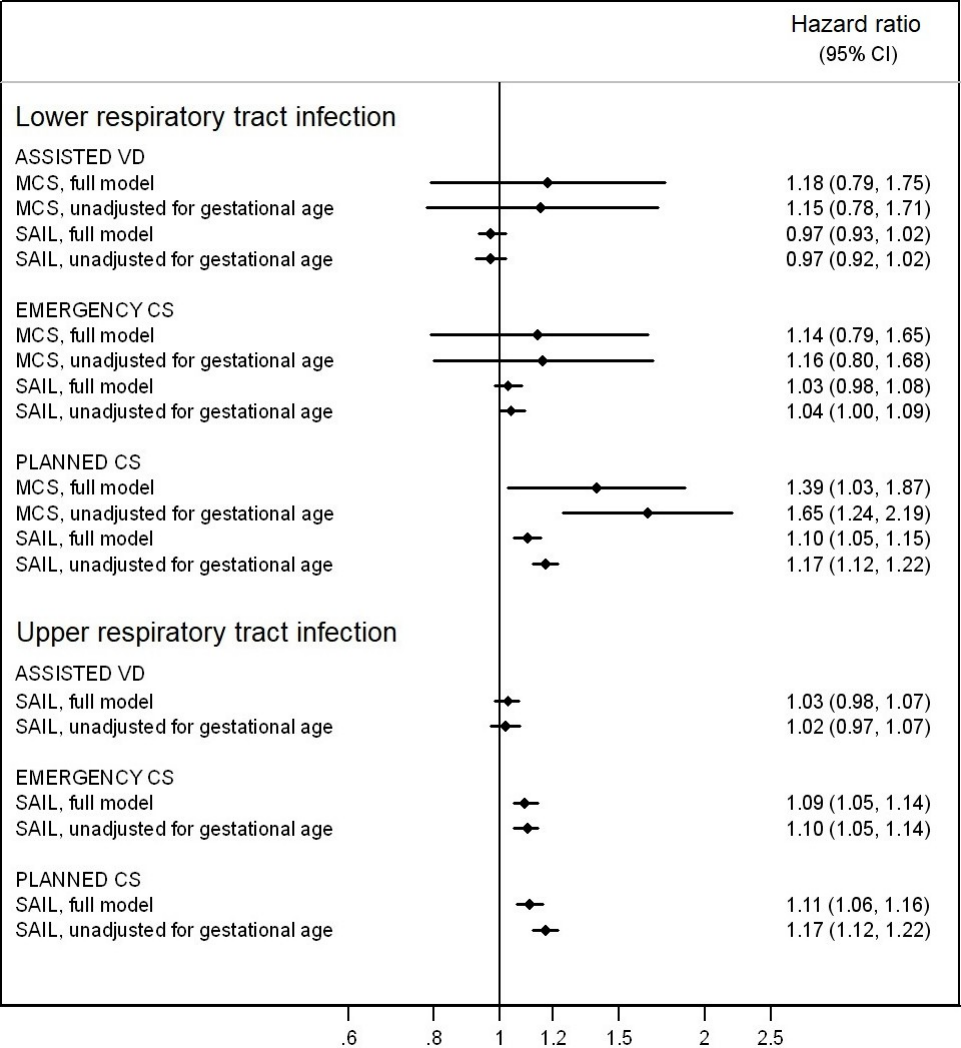
Hazard ratios of admission for LRTI and URTI, fully adjusted and unadjusted for gestational age at birth.

To obtain hazard ratios that also comprise the indirect effect of the planned caesarean through the earlier timing of the delivery (e.g. 37–38 weeks), final models were repeated without adjusting for gestational age. The size of effects found in assisted vaginal delivery and emergency caesarean were unchanged (Fig 2). However, in infants born by planned caesarean, the hazard ratio for LRTI increased from 1.39 to 1.65 in the MCS and from 1.10 to 1.17 in SAIL when gestational age was removed from the model. Likewise, the hazard ratio for URTI increased from 1.11 in the fully adjusted model to 1.17 in the model unadjusted for gestational age in SAIL.

## Discussion

Our study demonstrated that birth by planned caesarean section is an independent risk factor associated with 10–39% increase in risk for LRTI hospital admission during infancy. Our study further showed that birth by planned or emergency caesarean section is an independent risk factor associated with a 9–11% increase in risk for URTI admission. When the effect of gestational age at delivery is not controlled, but rather included in the overall effect arising from the plan to deliver by caesarean, the effect size found is stronger.

### Comparison with literature

Prior studies have also identified planned caesarean as a risk factor for LRTI, as described here. However, the evidence regarding emergency caesarean is less conclusive, with some studies finding no increased risk, as in our study, and others identifying emergency caesarean as a risk factor as well.

Large population-based studies from Australia concluded that both types of caesarean are risk factors for LRTI admission. A study from New South Wales [9] found an increased risk in the magnitude of 12–13% while a study from Western Australia [10] reported that elective caesarean is associated with 34% increased risk of admission and emergency caesarean with 20% elevated risk. Likewise, an international study pooling data from several high-income countries found 15% elevated risk of admission associated with elective caesarean and a slightly lower risk of 9% associated with emergency caesarean [13].

Other studies highlighted elective caesarean, but not emergency caesarean, as a risk factor for LRTI, similarly to our study. Studies conducted in Denmark [8, 11] and Western Australia [7] examined infant and child admissions for bronchiolitis or respiratory syncytial virus, which is responsible for most bronchiolitis cases [34]. These studies found an 11–29% increased risk of admission associated with elective caesarean, but no increased risk for emergency caesarean. Likewise, a study examining recurrent episodes of LRTIs during early childhood in a Norwegian birth cohort reported 19% increased risk (although not statistically significant) in the elective caesarean group, and no association in the emergency caesarean group [35].

We are aware of only a single study that had examined the association of mode of birth with severe URTIs using population data. Similarly to our findings, the study found 12% elevated risk for admission in children born by emergency caesarean and a slightly larger 18% increased risk in children born by elective caesarean. A smaller study followed up 334 children in Copenhagen and did not find an association between caesarean section (any type) and URTIs, although an association with LRTI was found [36]. Our results are therefore novel in highlighting hospital admission for URTIs as a potential adverse outcome of caesarean sections. Minor infectious episodes of the upper respiratory tract are generally more frequent than those of the lower respiratory tract [36] and admission for this indication during infancy is as common as admission for LRTI (both of a prevalence of 5.6–5.7% in our sample). Therefore, although the excess risk for URTI admission arising from the caesarean birth is small (10%), this evidence of a possible adverse outcome of caesarean section is important.

Some infants experiencing a hospital admission for respiratory infection will continue to have persistent episodes of infection or wheeze later in childhood. In certain cases, the admissions during infancy may be an early sign of subsequent asthma diagnosis [37]. Analogous to our findings, studies focusing on asthma have identified a 20% elevated risk in children born by caesarean, in particular when elective [11, 38].

Birth at every successive week earlier than 39 or 40 weeks is associated with increased risk of childhood infections [5, 15]. During the years when many of the children included in our study were born (particularly the MCS sample), a relatively high proportion of planned caesareans were carried out at early term gestation (54.2% in the MCS and 34.0% in SAIL). Notably, this proportion decreased in the UK in more recent years due to guidelines recommending to refrain from routinely scheduling caesareans for earlier than 39 weeks [39, 40]. Our results therefore demonstrate the added effect of the timing of delivery to that of the caesarean birth and the significance of reporting the long-term effects of planned caesarean unadjusted for gestational age.

### Strengths and limitations

The main strength of our study is the use of two complementary cohorts to examine the association of mode of birth with LRTIs. The MCS includes rich data which enabled us to include variables such as breastfeeding that have not been previously well accounted for. The SAIL cohort was large enough to allow detection of modest effect sizes and provided a platform to investigate admission for URTIs. A further strength is our focus on term-born neonates, since prematurity is likely a strong confounder, as it influences the choice of mode of birth and is a risk factor for poor respiratory health [5]. Moreover, the exclusion of ill neonates helps address bias from confounding by indication. Complications during pregnancy and birth may indicate a medically-assisted delivery and at the same time lead to complications in the neonate, which may predispose to later disease risk. Lastly, the definitions and validation of the exposure and outcome variables are also strengths of the study. The exposure was validated using an additional data source, therefore increasing confidence that planned and emergency caesareans were correctly classified. The outcome focused on infections that require hospital admission, which are not likely influenced by parental health-seeking behaviour.

Limitations of the study include the unavailability of information about URTI admissions in the MCS and examination of this outcome solely through the SAIL datasets. The SAIL cohort is limited in having only area-level information about socio-economic factors, crude data on breastfeeding and under measurement of variables such as maternal asthma, which was collected through diagnosis codes at delivery. For LRTI, the MCS is limited by measurement of this outcome through maternal report, although this might not be a serious limitation since it has been shown that mothers generally recall their child’s hospital admission well [41], particularly considering the short recall period of less than one year. Nevertheless, we cannot rule out some degree of misclassification between various respiratory diagnoses in our outcome measurements. Lastly, residual confounding is a possible source of bias in both cohorts since potentially important covariates such as fetal or maternal complications, prophylactic antibiotics given at birth and induction of labour, were unavailable [14, 42].

### Mechanisms

Several biological mechanisms could potentially explain how a caesarean birth might affect subsequent risk of respiratory infection. First, the hormones of labour and the physical forces of contractions which squeeze amniotic fluid out of the lungs of the fetus, both have a role in establishing normal pulmonary function in the newborn [43]. A birth where this hormonal secretion or physical force does not occur (i.e. caesarean) may result in retained lung fluids, which can lead to transient tachypnoea of the newborn or, in severe cases, respiratory distress syndrome [44]. These conditions are more likely following planned caesarean section, especially at earlier gestations, compared to other modes of birth. If the respiratory health of the neonate affects later susceptibility to lower respiratory tract infection, this may explain the results found in the current study. Second, infants born by caesarean section show altered microbiota colonization as they do not benefit from the natural transmission of microbes from their mother’s vaginal flora [45] and have higher abundancy of opportunistic pathogens common in the hospital environment [46]. There is also emerging evidence to suggest that differences in microbial composition are especially pronounced following an elective caesarean, perhaps since amniotic membranes are still intact prior to delivery [47, 48]. Although the gut microbiota is the body area most studied in relation to mode of birth, other areas such as the upper airways also exhibit delayed development and reduced abundancy of beneficial bacterial strains in infants born by caesarean [49]. Dysbiosis of the microbiota has been linked to various adverse health conditions, including respiratory infections [50, 51].

## Conclusion

Planned caesarean section is a small but important risk factor for hospital admission for lower respiratory infections during infancy. Both planned and emergency caesarean section may be small risk factors for admissions due to upper respiratory infections. The risk of both types of respiratory infection is further elevated in infants born by planned caesarean due to the indirect increased risk from birth at an earlier gestational week. Although the effect sizes are modest, these excess risks are important in view of the large number of caesareans carried out globally.

## Supporting information

S1 File (DOCX)
