# Correction: Caesarean section and severe upper and lower respiratory tract infections during infancy: Evidence from two UK cohorts

**DOI:** 10.1371/journal.pone.0248548

**Published:** 2021-03-16

**Authors:** The PLOS ONE staff

## Notice of republication

An incorrect version of the running short title was published in error. The publisher apologizes for the error. This article was republished on March 1, 2021, to correct for this error. Please download this article again to view the correct version.
