# Supplementary material for: Caesarean section and severe upper and lower respiratory tract infections during infancy: Evidence from two UK cohorts
Source: PLoS One. 2021 Feb 16;16(2):e0246832. doi: 10.1371/journal.pone.0246832 (PMC7886211; doi:10.1371/journal.pone.0246832)
Supplement: S1 File — (DOCX) [file pone.0246832.s001.docx]

S1 Box: Congenital anomalies and genetic abnormalities excluded from the cohorts

| SAIL* | | | MCS |
| --- | --- | --- | --- |
| ICD code | **System** | **Annotation** | **Maternal report** |
| Q00 | Nervous system | Anencephaly and similar malformations |  |
| Q01 | Nervous system | Encephalocele |  |
| Q03 | Nervous system | Congenital hydrocephalus |  |
| Q05 | Nervous system | Spina bifida |  |
| Q23.4 | Circulatory system | Hypoplastic left heart syndrome | Congenital heart disease, definite |
| Q20.3 | Circulatory system | Discordant ventriculoarterial connection |  |
| Q21.0 | Circulatory system | Ventricular septal defect |  |
| Q33.8 | Circulatory system | Other congenital malformations of lung (including Cystic adenomatoid malformation of lung) |  |
| Q60.1 | Urinary system | Renal agenesis, bilateral |  |
| Q79.3 | musculoskeletal system | Gastroschisis |  |
| Q79.0 | musculoskeletal system | Congenital diaphragmatic hernia |  |
| Q90 | Chromosomal abnormalities | Down Syndrome (Trisomy 21) | Chromosomal or genetic abnormalities |
| Q91 | Chromosomal abnormalities | Edwards' syndrome (Trisomy 18) and Patau's syndrome (Trisomy 13) |  |
| Q96 | Chromosomal abnormalities | Turner's syndrome (45X) |  |
|  |  |  | Other congenital abnormalities, major |

* Information from CARIS (Congenital Anomaly Register and Information Service for Wales)

S1 Text: Sensitivity analyses for handling missing values in SAIL

Complete case analyses were carried out using solely observations with complete data on all variables, including for smoking, breastfeeding and ethnicity.

Chained imputations (80 sets) for maternal smoking, breastfeeding and ethnicity were performed using the variables included in final adjusted model, with the addition of the relevant outcome variable [3]. Following imputation, the distribution of each imputed variable was compared to its original distribution in complete cases and found to be almost identical.

S2 Text: Sensitivity analyses using validated mode of birth observations in MCS and SAIL

We used an alternative source of information about mode of birth in order to validate this variable (S2 Box). For the MCS, we used linked hospital records from the cohort infant’s delivery episode. Consent to link these records was given by 90% of the MCS mothers [1] and a matching record was found for 76% of MCS infants. We retrieved OPCS codes from the delivery episode and, where available, information about method of delivery from the ‘maternity tail’ of hospital episode statistics [2]. The mother’s report of mode of birth was considered valid if it was in agreement with either the OPCS codes or the ‘maternity tail’ delivery variable when grouped into four categories.

For the SAIL cohort, we used information about mode of birth from the NCCHD, which was available for 59% of the sample. In the MCS, the mother’s report of mode of birth was found valid in 11,328 observations (95% of observations with linked delivery records). In the SAIL cohort, the OPCS codes were found valid in 218,203 observations (94% of observations with NCCHD delivery variable). The results of the Cox regression in the validated sub-samples were similar to the main findings (S3 Table).

S2 Box: Variables used in the MCS and SAIL cohorts for validation of mode of birth

| Mode of birth classification | MCS | | | SAIL | |
| --- | --- | --- | --- | --- | --- |
|  | **MCS Maternal report  (main variable)**  **N=15,580** | **Linked hospital delivery records**  **(validation variables)**  **N=11,909 (76%)** | | **OPCS codes  from hospital delivery record  (main variable)**  **N=392,145** | **Delivery method  from NCCHD  (validation variable)**  **N=231,232 (59%)** |
|  |  | **OPCS codes** | **Maternity tail** |  |  |
| Vaginal delivery | Normal delivery  Waterbirth | R24 Normal delivery  R23 Cephalic vaginal delivery with abnormal presentation of head at delivery without instrument | Spontaneous vertex  Spontaneous other cephalic | R24 Normal delivery R23 Cephalic vaginal delivery with abnormal presentation  of head at delivery without instrument | Spontaneous vertex  Spontaneous  other cephalic |
| Assisted vaginal delivery | Forceps Vacuum Assisted breech Other assisted delivery | R19 Breech extraction delivery R20 Other breech delivery R21 Forceps cephalic delivery R22 Vacuum delivery | Low forceps, not breech Other forceps, not breech Ventouse, vacuum extraction Breech Breech extraction | R19 Breech extraction delivery R20 Other breech delivery R21 Forceps cephalic delivery R22 Vacuum delivery | Low forceps, not breech Other forceps, not breech Ventouse Breech Breech extraction |
| Planned / Elective caesarean | Planned caesarean | R17 Elective caesarean delivery | Elective caesarean section | R17 Elective caesarean delivery | Elective caesarean section |
| Emergency caesarean | Emergency caesarean | R18 Other caesarean delivery | Emergency caesarean section | R18 Other caesarean delivery | Emergency caesarean section |
| Missing | Refuse to answer, not known, irrelevant response, non-codable | R25 Other method of delivery |  | R25 Other method of delivery | Other |

S1 Table: Characteristics of the MCS and SAIL cohorts

|  | MCS* n (%)**  N=15,580 | SAIL n (%)  N=392,145 |
| --- | --- | --- |
| *Socio-demographics* |  |  |
| Maternal age, mean (SD) | 28.8 (0.12) | 28.1 (0.01) |
| Marital status |  |  |
| Married | 9,219 (60.8) | – |
| Cohabiting | 3,740 (25.1) | – |
| Single | 2,621 (14.1) | – |
| Education level |  |  |
| Higher | 4,581 (33.1) | – |
| Intermediate | 2,220 (14.3) | – |
| Lower | 5,812 (37.7) | – |
| Overseas or other academic/vocational | 483 (2.5) | – |
| None of these | 2,459 (12.4) | – |
| Household socioeconomic class |  |  |
| Managerial/Professional | 6,098 (45.3) | – |
| Intermediate | 3,064 (19.8) | – |
| Routine/Manual | 5,463 (30.1) | – |
| Unemployed | 955 (4.6) | – |
| Area deprivation quintile |  |  |
| 1^st^ (most deprived) | – | 101,588 (25.9) |
| 2^nd^ | – | 85,664 (21.8) |
| 3^rd^ | – | 75,816 (19.3) |
| 4^th^ | – | 66,197 (16.9) |
| 5^th^ (least deprived) | – | 62,880 (16.0) |
| White ethnicity | 12,906 (87.3) | 319,308 (91.7) |
| *Maternal-perinatal* |  |  |
| Male | 7,897 (50.5) | 200,134 (51.0) |
| Firstborn | 6,403 (41.1) | 153,948 (42.1) |
| Maternal asthma | – | 21,676 (5.5) |
| Maternal atopy | 6,213 (42.3) | – |
| Diabetes | 249 (1.9) | 8,775 (2.2) |
| Hypertension | 968 (7.0) | 22,129 (5.6) |
| Birthweight in kg, mean (SD) | 3.45 (0.64) | 3.45 (0.48) |
| Gestational age |  |  |
| 37-38 (early term) | 3,149 (20.3) | 72,094 (18.4) |
| 39-40 (full term) | 8,358 (53.6) | 213,392 (54.6) |
| 41 (late term) | 3,482 (22.4) | 87,965 (22.5) |
| 42 (post term) | 559 (3.8) | 17,720 (4.5) |
| Maternal smoking |  |  |
| Non-smoker | 10,200 (66.4) | 121,582 (77.0) |
| Gave-up in pregnancy | 1,878 (12.6) | 5,771 (3.7) |
| Smoker | 3,497 (21.0) | 30,475 (19.3) |
| Any breastfeeding | 10,284 (69.9) | 193,752 (58.4) |

* Percentages in MCS are weighted

** All MCS and SAIL percentages are calculated from non-missing observations. Missing values are <10% of total observations for all variables apart from the following in SAIL: 234,317 (59.8%) for smoking, 60,163 (15.3%) for breastfeeding, 44,040 (11.2%) for ethnicity

S1 Figure: Frequency of hospital admissions (first admission) for LRTI by infant age

A. MCS cohort


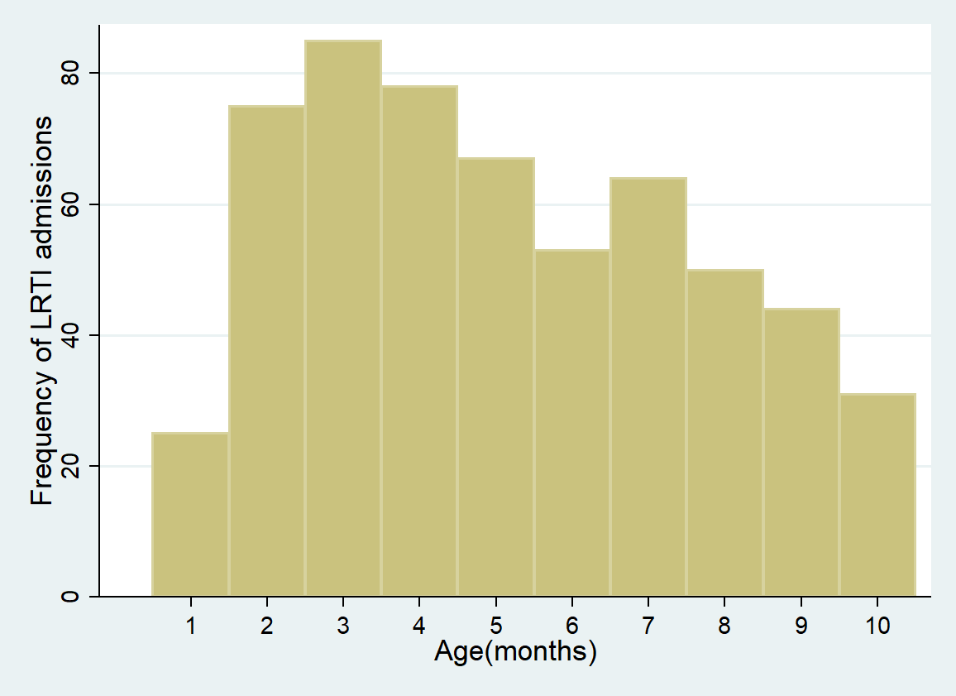


* follow-up complete until 8^th^ month only

B. SAIL cohort


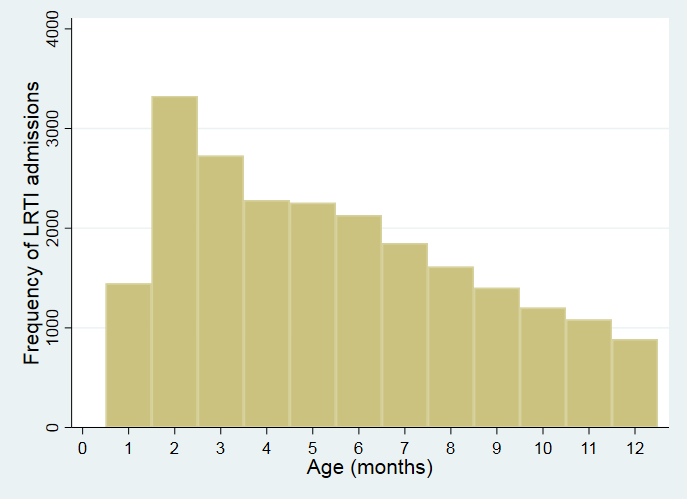


S2 Table: Adjusted hazard ratios for sensitivity analyses of complete cases and multiple imputation in the SAIL cohort compared to the main analyses

|  | Hospital admission for  Lower Respiratory Tract Infection  in SAIL | | | Hospital admission for  Upper Respiratory Tract Infection  in SAIL | | | |
| --- | --- | --- | --- | --- | --- | --- | --- |
|  | Main analysis  Adjusted* HR  (95% CI)  N=364,651 | Complete case analysis  Adjusted* HR  (95% CI)  N=124,037 | Multiple imputation  Adjusted* HR  (95% CI)  N=364,651 | | Main analysis  Adjusted** HR  (95% CI)  N=364,654 | Complete case analysis  Adjusted** HR  (95% CI)  N=124,037 | Multiple imputation  Adjusted** HR  (95% CI)  N=364,654 |
| VD | 1.00 | 1.00 | 1.00 | | 1.00 | 1.00 | 1.00 |
| Assisted VD | 0.97 (0.93, 1.02) | 0.93 (0.84, 1.02) | 0.98 (0.93, 1.03) | | 1.03 (0.98, 1.07) | 0.98 (0.90, 1.07) | 1.02 (0.98, 1.07) |
| Planned CS | 1.10 (1.05, 1.15) | 1.12 (1.04, 1.22) | 1.10 (1.06, 1.15) | | 1.11 (1.06, 1.16) | 1.10 (1.02, 1.19) | 1.11 (1.06, 1.16) |
| Emergency CS | 1.03 (0.98, 1.08) | 1.00 (0.92, 1.08) | 1.02 (0.98, 1.07) | | 1.09 (1.05, 1.14) | 1.11 (1.03, 1.20) | 1.09 (1.05, 1.14) |

* Adjusted for maternal age, area deprivation quintile, firstborn, smoking, maternal asthma, hypertensive conditions, infant’s sex, ethnicity, gestational age, birthweight, breastfeeding, season and year of birth

** Adjusted for maternal age, area deprivation quintile, firstborn, smoking, maternal asthma, infant’s sex, ethnicity, gestational age, birthweight, breastfeeding, season and year of birth

S3 Table: Adjusted hazard ratios for the association between validated mode of birth and LRTI and URTI during infancy: sensitivity analyses compared to main analyses

|  | Hospital admission for  Lower Respiratory Tract Infection | | | | Hospital admission for  Upper Respiratory Tract Infection | |
| --- | --- | --- | --- | --- | --- | --- |
|  | **MCS** | | **SAIL** | | **SAIL** | |
|  | Main analysis    Adjusted* HR  (95% CI)  N=15,531 | Sensitivity analysis  validated mode of birth  Adjusted* HR  (95% CI)  N=11,296 | Main analysis    Adjusted** HR  (95% CI)  N=364,651 | Sensitivity analysis  validated mode of birth  Adjusted** HR  (95% CI)  N=198,765 | Main analysis    Adjusted*** HR  (95% CI)  N=364,654 | Sensitivity analysis  validated mode of birth  Adjusted*** HR  (95% CI)  N=198,765 |
| VD | 1.00 | 1.00 | 1.00 | 1.00 | 1.00 | 1.00 |
| Assisted VD | 1.18 (0.79, 1.75) | 1.29 (0.82, 2.03) | 0.97 (0.93, 1.02) | 1.01 (0.94, 1.08) | 1.03 (0.98, 1.07) | 1.04 (0.97, 1.11) |
| Planned CS | 1.39 (1.03, 1.87) | 1.60 (1.15, 2.23) | 1.10 (1.05, 1.15) | 1.08 (1.01, 1.15) | 1.11 (1.06, 1.16) | 1.08 (1.01, 1.15) |
| Emergency CS | 1.14 (0.79, 1.65) | 0.99 (0.63, 1.56) | 1.03 (0.98, 1.08) | 1.01 (0.95, 1.08) | 1.09 (1.05, 1.14) | 1.11 (1.04, 1.18) |

* Adjusted for maternal age, firstborn, infant’s sex, maternal smoking, gestational age and breastfeeding per month of follow-up

**Adjusted for maternal age, area deprivation quintile, firstborn, smoking, maternal asthma, hypertensive conditions, infant’s sex,
ethnicity, gestational age, birthweight, breastfeeding, season and year of birth

*** Adjusted for maternal age, area deprivation quintile, firstborn, smoking, maternal asthma, infant’s sex, ethnicity, gestational age, birthweight, breastfeeding, season and year of birth

S4 Table: Adjusted hazard ratios for a sensitivity analysis excluding infants with history of perinatal infection in the MCS

|  | Hospital admission for  Lower Respiratory Tract Infection  in the MCS | |
| --- | --- | --- |
|  | Main analysis  Adjusted* HR  (95% CI)  N=15,531 | Sensitivity analysis  excluding perinatal infection**  Adjusted* HR  (95% CI)  N=14,090 |
| VD | 1.00 | 1.00 |
| Assisted VD | 1.18 (0.79, 1.75) | 1.04 (0.69, 1.58) |
| Planned CS | 1.39 (1.03, 1.87) | 1.40 (1.03, 1.89) |
| Emergency CS | 1.14 (0.79, 1.65) | 0.99 (0.66, 1.50) |

* Adjusted for maternal age, firstborn, infant’s sex, maternal smoking, gestational age, and breastfeeding per month of follow-up

** Perinatal infection ascertained from mother’s report of urinary tract infection during pregnancy, early rupture of amniotic membranes, other maternal infection during pregnancy or labour or infection or suspicion of infection in the neonate during the first week after birth

S5 Table: Adjusted hazard ratios for a sensitivity analysis using an extended definition of LRTI including wheezing of asthma in the MCS

|  | Hospital admission for  Lower Respiratory Tract Infection  in the MCS | |
| --- | --- | --- |
|  | Main analysis  Adjusted* HR  (95% CI)  N=15,531 | Sensitivity analysis  definition of LRTI  including ‘wheezing or asthma’  Adjusted* HR  (95% CI)  N=15,531 |
| Number of events | 574 | 710 |
| VD | 1.00 | 1.00 |
| Assisted VD | 1.18 (0.79, 1.75) | 1.05 (0.74, 1.49) |
| Planned CS | 1.39 (1.03, 1.87) | 1.37 (1.05, 1.78) |
| Emergency CS | 1.14 (0.79, 1.65) | 1.07 (0.73, 1.55) |

* Adjusted for maternal age, firstborn, infant’s sex, maternal smoking, gestational age, and breastfeeding per month of follow-up

S2 Figure: Frequency of hospital admissions (first admission) for URTI by infant age
in SAIL


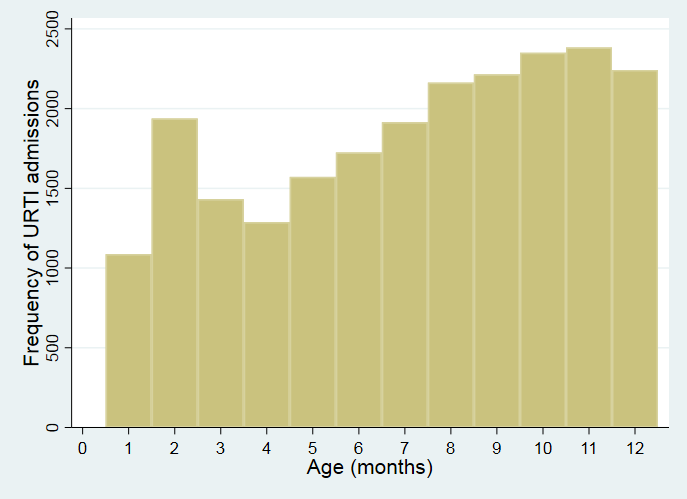


# References

1. Tate AR, Calderwood L, Dezateux C, Joshi H. Mother’s consent to linkage of survey data with her child’s birth records in a multi-ethnic national cohort study. Int J Epidemiol. 2006 Apr;35(2):294–8.

2. Knight HE, Gurol-Urganci I, Mahmood TA, Templeton A, Richmond D, van der Meulen JH, et al. Evaluating maternity care using national administrative health datasets: how are statistics affected by the quality of data on method of delivery? BMC Health Serv Res. 2013 Jan 30;13(1):200.

3. Royston P, White IR. Multiple Imputation by Chained Equations (MICE): Implementation in Stata. J Stat Softw. 2011;45(4).
